# Systematic Review on Infusion Reactions Associated with Chemotherapies and Monoclonal Antibodies for Metastatic Colorectal Cancer

**DOI:** 10.2174/157488412799218806

**Published:** 2011-02

**Authors:** Xue Song, Stacey R Long, Beth Barber, Cheryl A Kassed, Marcus Healey, Clare Jones, Zhongyun Zhao

**Affiliations:** 1 Healthcare, Thomson Reuters, Cambridge, MA, USA; 2 Global Health Economics, Amgen, Inc. Thousand Oaks, CA, USA; 3 Healthcare, Thomson Reuters, Washington, DC, USA;; 4 PRMA Consulting Ltd, Fleet, UK

**Keywords:** Chemotherapy, costs, infusion reaction, metastatic colorectal cancer, monoclonal antibody drugs, resource utilization.

## Abstract

**Objective:**

The objective of this systematic review is to summarize the literature to date on the rates of infusion reactions (IR) associated with chemotherapies and monoclonal antibody (mAb) drug therapies used for the treatment of metastatic colorectal cancer (mCRC) and the associated clinical and economic impact.

**Methods:**

This study searched Medline, Medline (R) In-Process, Embase and Cochrane Library databases for studies on IRs associated with chemotherapy and mAbs in mCRC patients from 2000-2011.

**Results:**

For chemotherapy, the incidence of IRs ranged from 0-71% for all grades and 0-15% for grade 3-4. Rates of all grade IRs associated with cetuximab ranged from 7.6-33% and grade 3-4 IR rates were 0-22%. Rates of all grade IRs associated with panitumumab ranged from 0-4% and rates of grade 3-4 IRs ranged from 0-1%. The overall rate of IRs associated with bevacizumab ranged from 1.6-11%, with a rate of 0-4% for grade 3-4 IRs. A range of 50-100% of patients with grade 3-4 IRs terminated chemotherapy, and 34-100% of cetuximab patients with grade 3-4 IRs discontinued cetuximab therapy. No data were reported for bevacizumab or panitumumab. Only one study evaluated the economic impact of IRs. The study compared cetuximab administrations without an IR to those with an IR requiring resource utilization and found that mean costs were $9308 and $1725 higher for those with an IR requiring an emergency room visit or hospitalization and for those with an IR requiring outpatient treatment, respectively.

**Conclusions:**

The incidence of IRs varies among different mAbs; and IRs may cause treatment disruption and require costly medical interventions.

## INTRODUCTION

Colorectal cancer (CRC) is the third most frequently diagnosed cancer and the third leading cause of cancer death in the USA. According to estimates based on the Surveillance, Epidemiology and End Results (SEER) database, about 20% of patients with CRC are diagnosed with metastasis [1], and 50% of patients treated for early stage CRC will eventually develop metastases [2]. The 5-year survival rate for patients with metastatic CRC (mCRC) is about 10% [1]. In Europe, CRC is the second most common form of cancer and also the second leading cause of death from cancer [3].

Recent advances in molecular oncology and an enhanced understanding of tumor cell signaling pathways have led to new targeted biologic therapies for mCRC that have translated into improvements in patient outcomes. Among the new agents are three monoclonal antibody (mAb) drugs: bevacizumab, a humanized mAb targeting vascular endothelial growth factor (VEGF); cetuximab and panitumumab, both targeting epidermal growth factor receptors (EGFR) with cetuximab being a chimeric while panitumumab being a fully human mAb. Progress towards improved outcomes is still ongoing. For example, identification of wild-type *KRAS* tumor status as a biomarker for benefit from anti-EGFR agents (cetuximab and panitumumab) represents an important step towards personalized treatment of mCRC [4-7].

Infusion reactions (IRs) have been documented with chemotherapies and mAbs that are administered intravenously [8,9]. The mechanism of IRs is not clearly understood and may vary between agents [8]. Chung and colleagues have showed that in most patients experiencing severe IRs to cetuximab, immunoglobulin E (IgE) antibodies against cetuximab were present in serum before therapy and the antibodies were specific for galactose-α-1,3-galactose [10]. Thus, it is important to realize that IRs can be both non-IgE-mediated and IgE-mediated reactions, and they are difficult to discriminate based on clinical presentation [11]. 

According to the National Cancer Institute Common Toxicity Criteria for Adverse Events v3.0 [12], grade 1 reactions generally do not require infusion interruption or intervention; grade 2 reactions are often managed by supportive therapy, infusion interruption or symptomatic treatment.High grade (3 or higher) IRs can be prolonged and may not respond rapidly to symptomatic medications, and can result in hospital events that require supportive patient care [13-15]. High grade symptoms include urticaria, nausea, vomiting, pruritus, bronchospasm, dyspnea and tongue swelling, which may progress to hypotension, respiratory arrest, and occasionally death. Although rare, deaths have resulted from severe IRs [14,16]. 

The objective of this systematic review is to summarize the literature to date on the rates of IRs associated with chemotherapies and mAb therapies used for the treatment of mCRC, and resource utilization and cost burden of IRs. 

## METHODS

This systematic search strategy was designed to identify studies (randomized controlled trials, prospective and retrospective studies), and systematic reviews reporting IRs. Systematic searches of the Medline, Medline (R) In-Process, Embase and Cochrane Library databases were performed to identify relevant articles published in English between 2000 and 2011. Non-systematic reviews, case reports, research briefs, letters, editorials, studies in animals and phase I and IIa studies were excluded. The systematic search was global in nature and was not restricted to specific countries.

Search terms included ‘infusion reaction’, ‘allergic reaction’, ‘hypersensitivity’, and ‘anaphylaxis’ combined with terms to identify articles relating to advanced or metastatic CRC. Treatments of interest included were fluorouracil, bevacizumab, irinotecan, oxaliplatin, cetuximab, panitumumab, capecitabine and regimens composed of combinations of these therapies. Pro-drugs were not considered treatments of interest. Articles in the following categories were excluded: cancers other than colorectal, non-advanced or metastatic cancer, adverse events not indicative of an IR, not treatment of interest, and study of other topics. 

## RESULTS

The initial systematic searches of the literature retrieved 6502 studies. After excluding duplicate articles and the removal of obviously irrelevant records, such as those for other cancers than mCRC, 733 studies remained for more detailed assessment. Following the first pass categorization, 196 studies were selected for further assessment. Only 14 records were rejected during the second pass categorization; 182 records went for abstract review. The abstract review excluded 37 records, leaving 145 studies for full article review. A further 70 records were rejected, which led to 74 articles for data extraction. An additional three studies were identified in a manual literature search. Consequently, there were a total of 77 studies that met all review criteria for the entire IR systematic review. Of these 77 studies, approximately 69% (53/77) of studies were prospective and 31% (24/77) were retrospective in nature; and 39 of them included monoclonal biologic therapies. 

## INCIDENCE RATES OF IRS

Package inserts and the 33 studies that report rates of IRs associated with chemotherapies, bevacizumab, cetuximab and panitumumab, are summarized in Table **1**. For chemotherapy treatments, the incidence of all grade IRs ranged from 0-71%; for grade 3-4 IRs the ranges were 0-15% [17-22]. The incidence of IRs was highest in regimens which included oxaliplatin. Few of the studies attributed the IRs to one particular drug. 

The incidence of IRs varies with the different mAbs (alone or with combination of chemotherapy), and in clinical trials and non-clinical trials [49].

### Bevacizumab

The overall rate (all grades) of IRs associated with the first infusion of bevacizumab, as described in the package insert, is less than 3% and severe reactions occurred in 0.2% of patients [23]. 

#### Clinical Trials

Tol and his colleagues conducted two phase III clinical trials in patients with mCRC treated with capecitabine, oxaliplatin and bevacizumab, with and without cetuximab. The 2008 clinical trial reported an overall IR rate of 11% and 3% grade 3-4 IRs in patients treated with capecitabine, oxaliplatin, and bevacizumab (without cetuximab) and in those patients received cetuximab the overall IR rate and grade 3-4 IR rate was 23% and 7%, respectively [24]. In the 2009 clinical trial, grade 3-4 IRs occurred in 4% of patients treated without cetuximab and 4.9% in those treated with cetuximab [25]. It is worth noting that all pivotal clinical trials for bevacizumab do not report information on IRs [50-52]. 

#### Non-Clinical Trials

Schwartzberg and colleagues retrospectively reviewed the charts from 19 community oncology centers to identify patients who had received mAb treatment either as monotherapy or in combination with chemotherapy, and who had documented evidence of a severe IR during therapy [14]. Of 76 identified IRs (total number of charts reviewed was not reported), five patients who had been treated with bevacizumab experienced infusion reactions, and all five were grade 3 reactions. However, the study was not designed to assess the incidence rate of mAb-related IRs, but rather to descriptively study the clinical care associated with the events. 

Computerized pharmacy records were used to identify all patients who received bevacizumab at one cancer center in a study by Reidy *et al.,* [26]. The center's adverse drug reaction reporting program was then used to identify any IRs related to bevacizumab with subsequent confirmation by medical record review. Six patients (1.6%) experienced minor IRs, five of whom were being treated for CRC (four with mCRC). No patients experienced severe IRs in that study. 

### Cetuximab 

As described in the package insert of cetuximab, IRs, which included pyrexia, chills, rigors, dyspnea, bronchospasm, angioedema, urticaria, hypertension and hypotension occurred in 15-21% of patients across studies [27]. Grade 3 and 4 IRs occurred in 2-5% of patients, and fatal outcomes associated with IRs were rare (<1 in 1000) [27]. Clinical trials found an overall rate of IRs that ranged from 7.6-33%, while the rate of grade 3-4 IRs ranged from 0-6%. Observational studies found an overall rate of IRs from 27-32%, and the rate of grade 3-4 IRs from 6.6-22%.

#### Clinical Trials

In a large randomized phase III trial of cetuximab plus best supportive care (n=287) versus best supportive care alone (n=285) for the treatment of patients with chemorefractory mCRC, all grades of IRs occurred in 20.5% of patients, and grade 3-4 IRs occurred in 4.5% of patients assigned to cetuximab. No patients randomized to best supportive care had any IRs [31]. In a large, randomized, open-label, multicenter study (n=1198) comparing 14-day cycles of cetuximab plus FOLFIRI and FOLFIRI alone, grade 3 or 4 infusion-related reactions were more frequent with cetuximab plus FOLFIRI than with FOLFIRI alone (2.5% vs 0%, p<0.001) [4]. In a multicenter, open-label, phase III study of patients with mCRC, Sobrero *et al.,* found that grade 3-4 IRs occurred to 1.4% of patients treated with cetuximab and 0.8% of those on irinotecan alone (n=648) [36]. 

In an open-label, randomized, multicenter phase II study comparing the efficacy and safety of cetuximab combined with FOLFOX4 versus FOLFOX4 alone in the first-line treatment of EGFR-expressing mCRC, 5% of patients treated with cetuximab + FOLFOX4 experienced grade 3 or 4 infusion-related reactions compared to 2% of patients treated with FOLFOX4 alone [53].

In a randomized phase II trial of cetuximab (with or without irinotecan) in 327 patients whose disease had progressed during or within 3 months after treatment with an irinotecan-based regimen, Cunningham and colleagues reported that 3.5% of patients on cetuximab monotherapy but no patients on cetuximab with irinotecan experienced severe IRs (grade 3 or 4) [29]. 

Additionally, five phase II clinical trials reported incidence rates of grade 3-4 IRs from 1-5% in patients with mCRC treated with cetuximab alone or cetuximab in combination with chemotherapies [28,32-34,38].

#### Non-Clinical Trials

Schwartzberg's retrospective chart review identified patients who had received cetuximab either as monotherapy or in combination with chemotherapy, and who had documented evidence of a severe IR during therapy [14]. Of 76 identified IRs, 24 occurred in patients receiving cetuximab- 58% were grade 3, 33% grade 4, and 8% died before transport to hospital emergency care. Another Schwartzberg *et al.,* study found that 32% of patients treated with cetuximab experienced grade 1 or 2 IRs, and 7% experienced grade 3-4 IRs [15].

Interestingly, one study based on a retrospective clinical review reported that rates of severe IRs are much higher in North Carolina and Tennessee. O'Neill and colleagues analyzed data from the records of 88 patients with a variety of tumor types (39 with CRC) who were treated with cetuximab in clinical trials in these two states. That analysis found an overall rate of 28% for all grade IRs and 22% for grades 3-4 IRs associated with cetuximab treatment [42]. 

Using a large national retrospective claims database, Foley *et al.,* identified 1122 patients with CRC treated with cetuximab in the period 2004-2006. Among them, 8.4% developed IRs that required medical intervention like emergency room (ER) visits, hospitalization or outpatient treatment [40]. 

#### Clinical Trials on Bevacizumab and Cetuximab Combination Therapy

Three clinical trials included patients treated with both bevacizumab and cetuximab. A phase II clinical trial (n=83) by Saltz *et al.,* found a 0% IR rate of grade 3 attributable to cetuximab [35], a phase III clinical trial (n=192) by Tol *et al.,* found a 23% of all grade IRs and 7% of grade 3-4 IRs [24], and another phase III clinical trial (n=366) by Tol *et al.,* found a 4.9% of grade 3-4 IRs [25]. No separate rates of IRs were reported for bevacizumab and cetuximab in the two clinical trials conducted by Tol *et al.,* for patients treated with both bevacizumab and cetuximab. 

#### Panitumumab 

According to the panitumumab product insert, 4% of patients experienced IRs, and grade 3-4 IRs occurred in approximately 1% of all patients [43]. Clinical trials found a rate of grade 3-4 IRs of 0-0.7%. There were no non-clinical trials that reported the IR rate of panitumumab.

#### Clinical Trials

Van Cutsem and colleagues conducted a large, multicenter, randomized phase III clinical trial of panitumumab plus best supportive care (n=231) versus best supportive care alone (n=232) for the treatment of patients with chemorefractory mCRC. No patients experienced grade 3-4 IRs in patients receiving panitumumab plus best supportive care or in patients receiving best supportive care alone [47].

In a large, open-label, multicenter, phase III clinical trial that compared the efficacy of panitumumab + FOLFOX4 with FOLFOX4 alone in patients with previously untreated mCRC, 0.6% (two out of 322 patients) of patients treated with panitumumab + FOLFOX4 had grade 3 IRs (no grade 4 IRs) versus 0% in those treated with FOLFOX4 alone [6]. A similar finding was observed for panitumumab in second-line treatment of mCRC. Peeters *et al.,* assessed the safety and efficacy of panitumumab + FOLFIRI versus FOLFIRI alone in a large, open-label, multicenter, phase III clinical trial, grade 3-4 IRs were seen in 0.7% and 0% in patients treated with panitumumab + FOLFIRI and those treated with FOLFIRI alone, respectively [7]. Additionally, an overall IR rate of 0-0.7% was also in four phase II clinical trials [44-46,48]. 

## CLINICAL AND ECONOMIC IMPACT OF IRS

The occurrence and management of severe or mild IRs can have significant clinical and economic impact on patients, caregivers, and oncology practices, particularly because the majority of cancer care is provided in outpatient community-based facilities [14,15,54,55]. 

### Clinical Impact

Treatment interruption or discontinuation may be required for patients experiencing IRs. This has significant implications for mCRC treatment because in many cases mAbs are administered to patients whose disease has progressed following chemotherapy and, therefore, had limited treatment options. 

A total of 16 studies reported treatment termination due to severe IRs. Treatment was terminated in the majority of patients who experienced grade 3 or 4 IRs in these studies. There were only six studies that reported that some patients remained on treatment after a grade 3 or 4 IR [14,18,35,40,53,56] (Table **2**). 

Eight studies reported discontinuation of cetuximab due to severe IRs. Among the three patients who developed cetuximab-related grade 3 IRs in a phase II study by Saltz *et al.*, two discontinued cetuximab [35]. In the Foley *et al.,* study, 68% of cetuximab patients with any IRs requiring medical intervention experienced treatment interruption, of which 34% permanently discontinued cetuximab. For patients who experienced IRs that required emergency room visits or hospitalization, 53% discontinued cetuximab permanently [40]. In the George *et al.,* study, all patients with severe IRs discontinued cetuximab permanently, with 80% of them switching to panitumumab [41]. In a phase II clinical trial on patients with mCRC treated with cetuximab plus capecitabine/irinotecan, all patients with grade 3-4 IRs discontinued treatment [28]. Across all seven cetuximab studies included in Table **3**, 34-100% of cetuximab users who experienced grade 3-4 IRs terminated cetuximab treatment. There were four studies that reported that some patients remained on cetuximab after a grade 3 or 4 IR [14,35,40,53].

Schwartzberg and colleagues’ study is the only one that reported treatment disruption with IRs associated with bevacizumab. Three out of the five patients who experienced severe IRs permanently discontinued bevacizumab treatment during this study [14]. 

No studies reporting treatment termination involving panitumumab were identified. In a phase II clinical trial, one out of 148 panitumumab users developed a severe IR after the second panitumumab infusion [45]. That reaction was managed with antihistamine and analgesic treatment, and the patient was able to continue therapy.

Many of the articles reported the modification of intervention dosage based on the protocol specified, but did not report the number of dose reductions and delayed doses in the final results of their studies. For cetuximab, one study reported dose reductions [40], and three studies reported dose delays or changes in the rate of infusion [36,40,62]. For panitumumab, only one study reported dose delay or changes because of IRs [47]. However, it is difficult to estimate the actual number of dose reductions and delays because of the paucity of specific data. 

### Resource Use

Seven studies reported hospitalization of patients for the treatment of IRs [14,18,35,40,41,56,63]. The proportion of patients experiencing IRs who were hospitalized varied from 7.7-39.4%. A prospective time and motion study of cancer patients with severe IRs in the USA found that 22% were hospitalized with a 4-day length of stay on average [15]. In addition, patients with IRs required between 31% and 80% additional staff time [15]. The percentage of patients with CRC hospitalized for IRs associated with cetuximab treatment varied from 14.3-39.4% [14,35,40,41] (Table **3**). No separate data exist that reported hospitalization rates for IRs associated with bevacizumab or panitumumab.

### Economic Impact 

Studies that examined the economic impact of IRs are very limited. At the time of this review, Foley *et al.,* was the only study that directly estimated the incremental healthcare costs associated with treatment of IRs in patients with CRC treated with cetuximab [40]. Using a US national claims data set, they estimated that the mean cost was $13,863 for cetuximab administrations with an IR requiring an emergency room visit or hospitalization and $6280 for those with an IR requiring outpatient treatment, compared to costs of $4555 for those without an IR [40]. 

### Impact on Patients, Clinicians and Caregivers

IRs impose significant burden on patients, healthcare providers and caregivers. This includes psychological impact and burden on patients and clinicians’ attitude towards treatment and economic burden on patients’ families including out-of-pocket expenses and travel time to the outpatient clinic and hospital [54,55]. IRs are emotionally stressful and disruptive for patients and their caregivers. During in-person interviews of 202 oncology nurses in the USA, 87% reported that both patients and clinicians feel ‘fear’ and ‘stress’ even with the occurrence of mild IRs. Grade 3 or 4 IRs were ‘very’ or ‘extremely’ disruptive for patients and disruptive to nurses 80% of the time [64]. O’Neil *et al.,*. noted that the experience of an IR can be traumatic for patients, family members, and the clinical staff managing these events [42]. 

## DISCUSSION

Although mCRC remains incurable, the introduction of mAb therapies has improved patient treatment outcomes. However, severe IRs can occur with these treatment options and their management can be challenging to patients and clinicians. 

This systematic review of the literature included 77 articles on IR rates resulting from the currently available chemo- and mAb therapies in patients with mCRC, and the burden of these IRs. For chemotherapy treatments, the incidence of IRs ranged from 0-71% for all grades and 0-15% for grade 3-4. The literature on cetuximab was most common with 17 studies published beyond those noted in the product’s package insert. However, there was considerable heterogeneity in rates of IR observed across these studies. Of the 17 studies, the overall rates of all IRs ranged from a low of 7.6% to a high of 33%, as compared to rates of 15-21% as noted in the cetuximab package insert. Rates of severe IRs (grade 3-4) ranged from a low of 0% to a high of 22% found by in O’Neil *et al.,* [42]. Findings for panitumumab were more homogenous, but relied on fewer studies. Seven studies were available for panitumumab, with severe IRs ranging from 0-0.7%, compared to 1% reported in the package insert. Only three studies [24-26] were available for bevacizumab beyond the trials noted in the package insert. Reidy *et al.,* reported that 1.6% of cases experienced mild IRs, similar to the <3% documented in the package insert data [26]. Two clinical trials by Tol *et al.,* found a much higher rate of IRs with bevacizumab. The first study reported 11% of all grade IRs and 3% grade 3-4 IRs [24], and the second reported 4% severe IRs in patients treated with bevacizumab, capecitabine and oxaliplatin [25]. 

Within the available literature, the rates of IR for panitumumab appeared relatively consistent with rates observed in its clinical trials (as reported in panitumumab package insert). Conversely, there are substantial variations in the incidence rates of IRs associated with cetuximab included in the cetuximab product insert and those reported in various studies. However, most of the non-clinical trials were small studies. Some studies have found that the incidence of IRs varies between US geographic regions [43,65]. Atopic history and residence in the middle Southern region of the US have been associated with high incidence of severe IRs [40,56]. A relationship between prior allergy history and IRs was also noted, suggesting a pre-existing IgE-based immune reaction directed at the therapeutic antibody [10,11]. Foley *et al.,* found that residence in a state with high levels of pollen was associated with a higher likelihood of having an IR requiring medical intervention among patients with CRC treated with cetuximab [40]. While the prescribing information for cetuximab indicates that 90% of severe IRs occurred during the initial cetuximab administration, Needle reported that 33% of patients with severe IRs experienced events after their second dose of cetuximab [66]. Lenz also noted that 10-30% of IRs to mAbs are delayed and occur in later infusions [9].

There were also studies that reported findings on the clinical and financial impact of IRs, and many of these studies were related to the cetuximab treatment. In the majority of these studies, if patients experienced a grade 3 or 4 IR, treatment was terminated. The rate of discontinuation of therapy following a severe IR ranged from 34% to 100% - although the sample sizes from which these rates were determined were small. The claims database study by Foley *et al.,* found that compared to those without IRs, patients requiring outpatient management of an IR had approximately $1725 in incremental costs and those who had ER visits or were hospitalized due to their IRs had incremental costs of $9308.

Low grade reactions can often be managed by supportive therapy, infusion interruption or symptomatic treatment. Some patients can be re-challenged and remain on the same treatment after low grade reactions [9]. High grade reactions may demand immediate treatment interruption, modification or discontinuation, and can result in ER visits and hospital events that require supportive patient care [13-15,28,35,40,41]. 

Interpretation of results from this systematic review of the literature on IRs needs to take its limitations into consideration. The incidences of IR described in this review showed some heterogeneity among the included studies. This may reflect differences in definition of IRs, sample sizes, number of patients treated, study design, patient characteristics, concomitant chemotherapies, or many other factors among these studies. Particularly, most of non-clinical trials had small sample size and the estimates of IR rates were less reliable; thus, the interpretation of findings based on those studies needs to be cautious. 

## SUMMARY

There were relatively more published studies reporting IRs associated with the use of cetuximab and some of non-clinical trials reported higher rates of IRs in patients with mCRC treated with cetuximab than that was listed in the package insert. In contrast, the rates of IRs for panitumumab appear to be consistent with clinical trial findings, although relatively fewer studies have been published. Studies to date have also indicated that IRs often cause treatment disruption and require costly medical interventions. 

## Figures and Tables

**Table 1.** IR Rates Reported in Published Studies

Study	Year	Study Design: Treatment	Number of Patients	Overall IR Rate	Severe IR Rate
**Chemotherapy**					
Boige *et al.*, [22]	2008	Prospective: FOLFOX	44	23%	9%
Hsuen *et al.*, [19]	2003	Retrospective: FOLFOX4	47	11%	11%
Ichikawa *et al.*, [18]	2009	Retrospective record review: FOLFOX4 and FOLFOX6	105	25.7%	5.7%
Li *et al.*, [21]	2010	Phase II trial: XELOX	124	10.5%	0%
Neyns *et al.*, [20]	2006	Prospective: oxaliplatin and L-folinic acid-modulated 5-fluorouracil	9	11%	11%
Suenaga *et al.*, [17]	2008	Retrospective analysis of a single institution: FOLFIRI, FOLFOX4	47	0-71%	0-15%
**Bevacizumab **					
Bevacizumab package insert [23]	2009			<3%	0.2%
***Clinical Trials***					
Tol *et al.*, [24]	2008	Phase III trial: capecitabine, oxaliplatin and bevacizumab	197	11%	3%
Tol *et al.*, [25]	2009	Phase III trial: capecitabine, oxaliplatin and bevacizumab	366	Not reported	4%
***Non-Clinical Trials***					
Reidy *et al.*, [26]	2007	Retrospective observational: bevacizumab at 5mg/kg over 10 minutes	370	1.6%	0%
**Cetuximab**					
Cetuximab package insert [27]	2009			15-21%	2-5%
***Clinical Trials***					
Cartwright *et al.*, [28]	2008	Phase II trial : cetuximab with capecitabine and irinotecan	69	Not reported	4%
Cunningham *et al.*, [29]	2004	Phase II trial: cetuximab monotherapy	115	Not reported	3.5%
Cunningham *et al.*, [29]	2004	Phase II trial: cetuximab with irinotecan	212	Not reported	0%
Folprecht *et al.*, [30]	2006	Phase I/II: cetuximab with irinotecan/low dose 5-FU/folinic acid	6	33%	0%
Folprecht *et al.*, [30]	2006	Phase I/II: cetuximab with irinotecan/high dose 5-FU/folinic acid	15	13%	0%
Jonker *et al.*, [31]	2007	Phase III trial: cetuximab with best supportive care	287	20.5%	4.5%
Lenz *et al.*, [32]	2006	Phase II trial: cetuximab	346	7.6%	1.7%
Ocvirk *et al.*, [33]	2010	Phase II trial: cetuximab with FOLFOX6	77	Not reported	6%
Ocvirk *et al.*, [33]	2010	Phase II trial: cetuximab with FOLFIRI	74	Not reported	1%
Saltz *et al.*, [34]	2004	Phase II trial: cetuximab	57	Not reported	5%
Saltz *et al.*, [35]	2007	Phase II trial: cetuximab, bevacizumab, and irinotecan	43	Not reported	0%
Saltz *et al.*, [35]	2007	Phase II trial: cetuximab and bevacizumab, alone	40	Not reported	0%
Sobrero *et al.*, [36]	2008	Phase III trial: cetuximab with irinotecan	648	Not reported	1.4%
Soulgoulakos *et al.*, [37]	2007	Phase II trial: cetuximab with capecitabine and oxaliplatin	40	15%	2.5%
Wierzbicki *et al.*, [38]	2011	Phase II trial: cetuximab	85	Not reported	1.2%
***Non-Clinical Trial***						
Bachet *et al.*, [39]	2007	Retrospective record based: cetuximab with or without irinotecan, FOLFIRI	105	Not reported	≥6.6%
Foley *et al.*, [40]	2010	Large US national claims data: cetuximab with and without chemotherapy	1122	8.4% (requiring medical intervention)		
George *et al.*, [41]	2010	Retrospective cohort: cetuximab with or without chemotherapy	30	27%	17%
O'Neil *et al.*, [42]	2007	Retrospective clinical record review: cetuximab with unspecified chemotherapy	88	28%	22%
Schwartzberg *et al.*, [15]	2009	Prospective multi-center time and motion study: cetuximab	71	32%	7%
**Bevacizumab + cetuxmiab**						
Tol *et al.*, [24]	2008	Phase III trial: capecitabine, oxaliplatin and bevacizumab with cetuximab	192	23%	7%
Tol *et al.*, [25]	2009	Phase III trial: capecitabine, oxaliplatin and bevacizumab with cetuximab	366	Not reported	4.9%
**Panitumumab**						
Panitumumab package insert [43]	2010			4%	1%
*Clinical Trials*						
Berlin *et al.*, [44]	2007	Phase II trial: panitumumab with IFL	19	0%	0%
Berlin *et al.*, [44]	2007	Phase II trial: panitumumab with FOLFIRI	24	0%	0%
Douillard *et al.*, [6]	2010	Phase III trial: panitumumab with FOLFOX4	322	Not reported	0.6%
Hecht *et al.*, [45]	2007	Phase II trial: panitumumab	148	0.7	0.7%
Muro *et al.*, [46]	2009	Phase II trial: panitumumab	52	0%	0%
Peeters *et al.*, [7]	2010	Phase III trial: panitumumab with FOLFIRI	539	Not reported	0.4%
Van Cutsem *et al.*, [47]	2007	Phase III trial: panitumumab with best supportive care	229	0.4%	0%
Van Cutsem *et al.*, [48]	2008	Phase III trial: panitumumab	176	0.6%	0%

IR, infusion reaction; CAPOX, capecitabine + oxaliplatin; ILF: irinotecan, bolus 5-FU, and leucovorin; FOLFIRI, infusional 5-FU/LV + irinotecan; FOLFOX4, 5-FU/LV + oxaliplatin; mFOLFOX6, modified FOLFOX6; IRINOX, irinotecan + oxaliplatin; XELIRI, Xelox (capecitabine) + irinotecan; XELOX, Xeloda (capecitabine) + oxaliplatin.

**Table 2.** Studies with Reported Treatment Terminations as a Result of Severe Infusion Reactions

Author	Year	Grade of IRs	Treatment	Patients with IRs (%)	Treatment Terminations among those with IRs (%)
**Chemotherapy**					
Becouarn *et al.*, [57]	2007	Grade 4	IRINOX	3%	100%
Desai *et al.*, [58]	2005	Grade 3	Cyclosporine, irinotecan and 5-FU	6%	100%
Hsuen *et al.*, [19]	2003	Grade 3	FOLFOX4	11%	100%
Ichikawa *et al.*, [18]	2009	Grade 3-4	FOLFOX4 or Modified FOLFOX6	6%	50%
Kalofonos *et al.*, [59]	2006	Grade 5	FOLFOX	1%	100%
Matsumoto *et al.*, [56]	2008	Grade 3-4	Modified FOLFOX6	4%	60%
Seki *et al.*, [60]	2009	Grade 3	5FU followed by FOLFOX4 or modified FOLFOX6	5%	100%
Shibata *et al.*, [61]	2009	Grade 3	FOLFOX4	4%	100%
**mAbs**					
Bachet *et al.*, [39]	2007	Grade 3-4	Cetuximab	7%	100%
Bokemeyer *et al.*, [53]	2009	Grade 3-4	Cetuximab + FOLFOX4	5%	88%
Cartwright *et al.*, [28]	2008	Grade 3-4	Cetuximab + XELIRI	4%	100%
Foley *et al.*, [40]	2010	Grade 3-4	Cetuximab with or without chemotherapy	8%	34%
George *et al.*, [41]	2010	Grade 3	Cetuximab with or without chemotherapy	10%	100%
George *et al.*, [41]	2010	Grade 4	Cetuximab With or without chemotherapy	7%	100%
Saltz *et al.*, [34]	2004	Grade 3-4	Cetuximab	5%	67%
Schwartzberg *et al.*, [14]	2008	Grade 3-4	Cetuximab	NA	82%
Schwartzberg *et al.*, [14]	2008	Grade 3	Bevacizumab	NA	60%
Sobrero *et al.*, [36]	2008	Grade 3-4	Cetuximab + Irinotecan	1%	100%

FOLFOX4, 5-FU/LV + oxaliplatin; mFOLFOX6, modified FOLFOX6; IRINOX, irinotecan + oxaliplatin; XELIRI, Xelox (capecitabine) + irinotecan.

**Table 3.** Infusion Reactions Requiring Hospitalization

Author	Year	Treatment	Grade of Reaction	Incidence of Reactions	Of Patients who had IRs, % whom Required Hospitalization
**Chemotherapy**					
Brandi *et al.*, [63]	2003	FOLFOX 4	All grades	18%	10%
Ichikawa *et al.*, [18]	2009	FOLFOX4 or modified FOLFOX6	Grade 3-4	6%	16.7%
Matsumoto *et al.*, [56]	2008	FOLFOX 6	Grades 1-4 Grades 3-4	19.9% 4.5%	7.7% 20%
**mAbs**					
Foley *et al.*, [40]	2010	Cetuximab	IRs requiring medical intervention	8.4%	39.4%
George Jr *et al.*, [41]	2010	Cetuximab +/- chemotherapy	Grades 2-4	27%	14.3%
Saltz *et al.*, [35]	2004	Cetuximab	Grade 3-4	5.3%	33%
Schwartz *et al.*, [14]	2008	cetuximanb, bevacizumab	Grade 3 or higher	Not reported	22%
